# An Analysis of the Potential Relationship of Triglyceride Glucose and Body Mass Index With Stroke Prognosis

**DOI:** 10.3389/fneur.2021.630140

**Published:** 2021-04-22

**Authors:** Zongyi Hou, Yuesong Pan, Yindong Yang, Xiaofan Yang, Xianglong Xiang, Yilong Wang, Zixiao Li, Xingquan Zhao, Hao Li, Xia Meng, Yongjun Wang

**Affiliations:** ^1^Department of Neurology, Hongqi Hospital, Mudanjiang Medical University, Mudanjiang, China; ^2^Heilongjiang Key Laboratory of Ischemic Stroke Prevention and Treatment, Mudanjiang, China; ^3^Department of Neurology, Beijing Tiantan Hospital, Capital Medical University, Beijing, China; ^4^China National Clinical Research Center for Neurological Diseases, Beijing Tiantan Hospital, Capital Medical University, Beijing, China; ^5^Center of Stroke, Beijing Institute for Brain Disorders, Beijing, China; ^6^Beijing Key Laboratory of Translational Medicine for Cerebrovascular Disease, Beijing, China

**Keywords:** triglyceride glucose index, stroke, prognosis, body mass index, insulin resistance 3

## Abstract

**Background:** The inverse association between obesity and outcome in stroke patients (known as the obesity paradox) has been widely reported, yet mechanistic details explaining the paradox are limited. The triglyceride glucose (TYG) index has been proposed as a marker of insulin resistance. We sought to explore possible associations of the TYG index, body mass index (BMI), and stroke outcome.

**Methods:** We identified 12,964 ischemic stroke patients without a history of diabetes mellitus from the China National Stroke Registry and classified patients as either low/normal weight, defined as a BMI <25 kg/m^2^, or overweight/obese, defined as a BMI ≥ 25 kg/m^2^. We calculated TYG index and based on which the patients were divided into four groups. A Cox or logistic regression model was used to evaluate the association between BMI and TYG index and its influence on stroke outcomes, including stroke recurrence all-cause mortality and poor outcome (modified Rankin Scale score of 3–6) at 12 months.

**Results:** Among the patients, 63.3% were male, and 36.7% were female, and the mean age of the patient cohort was 64.8 years old. The median TYG index was 8.62 (interquartile range, 8.25–9.05). After adjusting for multiple potential covariates, the all-cause mortality of overweight/obese patients was significantly lower than that of the low/normal weight patients (6.17 vs. 9.32%; adjusted hazard ratio, 0.847; 95% CI 0.732–0.981). The difference in mortality in overweight/obese and low/normal weight patients with ischemic stroke was not associated with TYG index, and no association between BMI and TYG index was found.

**Conclusion:** Overweight/obese patients with ischemic stroke have better survival than patients with low/normal weight. The association of BMI and stroke outcome is not changed by TYG index.

## Introduction

Stroke is a leading cause of death and disability worldwide (1). Obesity is known to raise the risk of stroke (2). However, numerous reports have shown an inverse association between obesity and outcome in stroke patients, which is in contrast to the general population and is known as the stroke-obesity paradox (3–5). Although it is unknown if the stroke-obesity paradox is related to insulin sensitivity/resistance (IR), IR has been theorized to contribute to this phenomenon (6).

IR is a physiologic state in which a normal amount of insulin produces a subnormal physiologic response (7). IR is closely associated with obesity (8) and is an independent risk factor for mortality and major disability after stroke (9–11). Several mechanisms have been proposed to explain this association, including enhanced local inflammatory and prothrombotic responses and accelerated atherosclerotic development (12). The gold standard for measuring IR is the euglycemic hyperinsulinemia clamp (13). However, this method is difficult to apply in large population studies and clinical settings because of its costly, time-consuming and complex nature. In response to this limitation, large-scale epidemiological studies have resorted to the homeostasis model assessment of IR (HOMA-IR) index (9, 14), while the application of HOMR-IR is limited by the need for the fasting immunoreactive insulin.

A potential surrogate method of measuring IR has been developed, known as the triglyceride glucose (TYG) index (15). TYG index can be used in large-scale observational and/or interventional cohorts by comparing with the hyperinsulinemia-euglycemic clamp (16). Previous studies have shown that TYG index is positively correlated with ischemic stroke, and ischemic stroke patients with a high TYG index have a higher risk of dying or poorer outcome (17). Therefore, the aim of our study is to evaluate if the association between body mass index (BMI) and stroke outcomes is modified by TYG index.

## Methods

### Study Population

**Figure 1:**
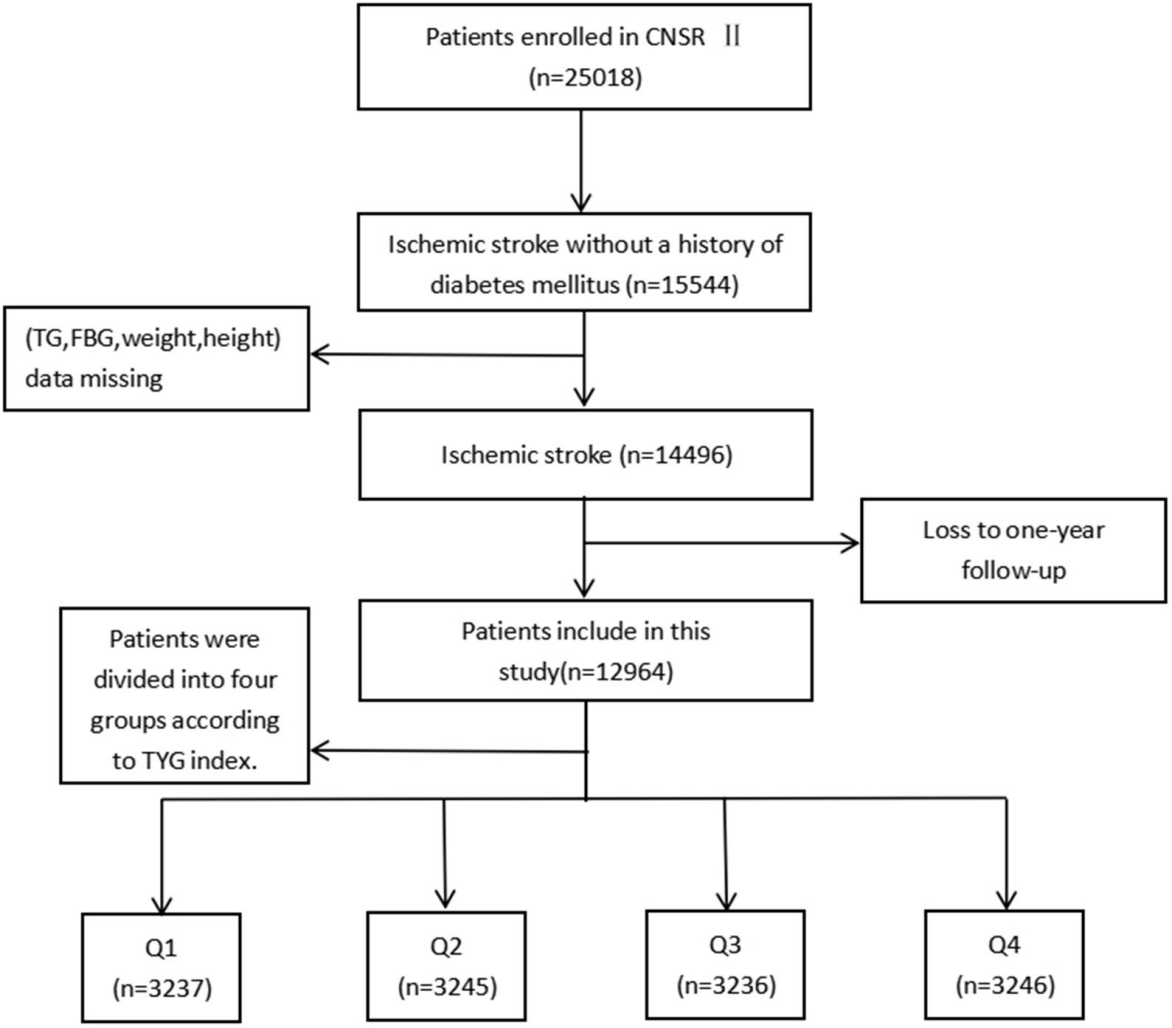
Patients selection flow diagram.

Launched in 2012 by the Chinese Ministry of Health, the China National Stroke Registry II (CNSR II) is a prospective cohort study conducted nationwide aimed at establishing a reliable national stroke database for evaluating stroke care delivery in clinical practice (18). The criteria for site selection in China National Stroke Registry I (CNSR I) have been previously published (19) and were replicated in the CNSR II study. Patient's included in the study must have met the following criteria: age ≥ 18 years old, diagnosed within 7 days of the index event of ischemic stroke, transient ischemic attack (TIA), spontaneous intracerebral hemorrhage, or subarachnoid hemorrhage confirmed by brain imaging, or direct hospital admission from a physician's clinic or emergency department. Of the 25,018 patients in the CNSR II, 15,544 were diagnosed with ischemic stroke without a history of diabetes mellitus. Ultimately, 12,964 were analyzed after excluding patients without necessary clinical data including weight, height, fasting triglyceride or fasting glucose at admission (*n* = 848), as well as those who were lost to follow-up at 12 months (*n* = 1,732; Figure 1).

The study was approved by the central Institutional Review Board at Beijing Tiantan Hospital and prior to the enrollment, all patients were provided written informed consent.

### Data Collection and Management

Patient baseline data were collected by trained research coordinators at each study center including basic patient characteristics, laboratory data, vascular risk factors, prior diagnoses and treatments. Vascular risk factors included stroke history, diabetes mellitus, hypertension, coronary heart disease, atrial fibrillation and prior/current smoking history. Past medication history included antihypertensive, antiplatelet drugs, anticoagulation, lipid-lowing, and hypoglycemic drugs.

BMI was measured by trained nurses at each clinical center at the time of patient enrollment. The delineation between low/normal and overweight/obese patients was set at 25 kg/m^2^, based on World Health Organization standards (20). Fasting blood samples were collected for all participants within 24 h of admission. Using an automated enzymatic method, triglyceride and glucose levels were tested at each research center. TYG index was calculated as ln[fasting triglycerides (mg/dl) × fasting glucose (mg/dl)/2] (21).

### Patient Follow-Up and Outcome Assessment

The primary outcomes of this study were stroke recurrence, all-cause mortality, and modified Rankin Scale (mRS) score, which were obtained by trained research coordinators that were blinded to the patients' baseline characteristics. Instances of recurrent stroke included ischemic stroke, intracranial hemorrhage, and subarachnoid hemorrhage. Poor functional outcomes were defined as a modified Rankin Scale score of 3–6. Modified Rankin Scale scores range from 0 (no symptoms) to 6 (death). At 3, 6, and 12 months after stroke, trained research personnel contacted patients by telephone following standard scripts, and collect information on death, stroke recurrence, and disability. During the follow-up, to ensure reliable diagnosis, stroke recurrences associated with rehospitalization were sourced to the corresponding hospitals. Adjudication was performed by the research coordinators together with the principal investigator in case of a suspected recurrent cerebrovascular event without hospitalization. Follow-up procedures for this study performed as previously described (22).

### Statistical Analysis

Continuous variables are presented as mean ± standard deviation or median with interquartile range (IQR). Categorical variables are presented as frequencies and percentages. We used a student's *t*-test to compare groups with normally distributed parameters, a Wilcoxon test for non-normally distributed parameters, and a χ^2^ test for categorical variables. A Cox proportional hazard analysis was conducted to estimate the hazard ratio (HR) and the 95% confidence interval (CI) of death and stroke recurrence. Odds ratios (OR) with 95% CIs were calculated for poor functional outcomes at 12 months and were estimated using a logistic regression model. Adjusted variables for HR with 95% CIs were sex, age, NIHSS score at admission, IV thrombolytic administration, prior/current smoking history, medical history (i.e., myocardial infarction, atrial fibrillation, hypertension, hyperlipidemia), medications (from the previously listed categories), and laboratory examination (i.e., total cholesterol, high density lipoprotein, low density lipoprotein). The impact of BMI and IR status on the risk of developing poor outcomes was also tested using Cox or logistic regressions. Kaplan-Meier analyses were used to assess survival from time of initial diagnosis until stroke recurrence during the 12-month follow-up period and a log-rank test was used to compare groups. We also examined the potential impact of age on the association between BMI and outcomes by separating patients into two groups using a cutoff of ≥65 years old. We assessed interactions between weight status in each age category using weight status × age category in multivariable models.

Statistical analyses were performed using SAS software (version 9.4, SAS Institute Inc, Cary, NC). Results were deemed statistically significant at a two-sided (*p* < 0.05).

## Results

### Baseline Patient Characteristics

Twelve thousand nine hundred sixty-four patients were included in the study, with an average age of 64.83 ± 12.2 years old and the gender distribution was 65.17% male and 34.83% female. The median TYG index was 8.62 (IQR, 8.25–9.05). We separated patients into four groups based on TYG index. Other baseline patient characteristics are summarized in Table 1. In comparing to low/normal patients, obese/overweight patients were typically younger, more likely to have a history of hypertension and received the treatment for hypertension prior to admission. Within the overweight/obese cohort, patients with a high TYG index were more likely to have a history of hyperlipidemia. Significant differences in the sex, NIHSS score, and history of anticoagulation therapy were also observed within groups.

**Table 1:** Baseline characteristics of patients stratified by TYG index and BMI status.

**Characteristic**	**TYG Q1(*****n=*** **3,237)**			**TYG Q2(*****n=*** **3,245)**			**TYG Q3(*****n*** **=** **3,236)**			**TYG Q4(*****n*** **=** **3,246)**		
	**BMI <25 kg/m^**2**^**	**BMI≥25 kg/m^**2**^**	***P-*value**	**BMI <25 kg/m^**2**^**	**BMI≥25 kg/m^**2**^**	***P-*value**	**BMI <25 kg/m^**2**^**	**BMI≥25 kg/m^**2**^**	***P-*value**	**BMI <25 kg/m^**2**^**	**BMI≥25 kg/m^**2**^**	***P-*value**
Age (years) mean ± SD	68.28 ± 12.09	64.70 ± 13.08	<0.0001	66.32 ± 11.8	64.21 ± 12.44	<0.0001	65.19 ± 12.01	61.83 ± 11.83	<0.0001	63.4 ± 11.1	61.0 ± 11.4	<0.0001
Male, n (%)	1,836 (70.79)	425 (65.38)	0.0055	783 (33.66)	353 (38.41)	0.0106	1,338 (64.36)	772 (62.40)	0.2678	1204 (62.22)	814 (62.09)	0.9392
NHISS score median (IQR)	4 (2–7)	3.5 (2–6)	0.0043	4 (2–7)	4 (2–6)	0.4723	4 (2–7)	4 (2–6)	0.0557	4 (2–7)	4 (2–6)	0.0029
thrombolysis (%)	54 (2.09)	3 (0.46)	0.0048	36 (1.55)	15 (1.63)	0.8616	19 (0.91)	26 (2.25)	0.0019	35 (1.81)	14 (1.07)	0.0894
Prior/current smoker (%)	1,257 (48.59)	302 (46.46)	0.3318	1,070 (46.00)	387 (42.11)	0.0447	984 (47.33)	521 (45.03)	0.2086	887 (45.84)	595 (45.39)	0.7986
**Medical history (%)**												
Myocardial infarction	57 (2.20)	7 (1.08)	0.0652	48 (2.06)	24 (2.61)	0.3397	37 (1.78)	29 (2.51)	0.1610	38 (1.96)	26 (1.98)	0.9689
Atrial fibrillation	245 (9.47)	65 (10.00)	0.6817	148 (6.36)	75 (8.16)	0.0681	148 (7.12)	57 (4.93)	0.0141	115 (5.94)	68 (5.19)	0.3593
Hypertension	1,382 (53.42)	408 (62.77)	<0.0001	1,390 (59.76)	626 (68.12)	<0.0001	1,270 (61.09)	837 (72.34)	<0.0001	1201 (62.07)	945 (72.08)	<0.0001
Hyperlipidemia	203 (7.85)	61 (9.38)	0.2004	199 (8.56)	103 (11.21)	0.0191	189 (9.09)	136 (11.75)	0.0157	216 (11.16)	184 (14.04)	0.0146
**Mediation History Prior to Stroke Hospitalization (%)**												
Antihypertensive drugs	904 (34.84)	280 (43.08)	<0.0001	886 (38.09)	433 (47.12)	<0.0001	841 (40.45)	576 (49.78)	<0.0001	800 (41.34)	620 (47.29)	0.0008
Antiplatelet drugs	456 (17.63)	128 (19.69)	0.2208	413 (17.76)	187 (20.35)	0.0865	330 (15.87)	215 (18.58)	0.0484	304 (15.71)	220 (16.78)	0.4160
Anticoagulation drugs	37 (1.43)	2 (0.31)	0.0190	25 (1.07)	14 (1.52)	0.2907	15 (0.72)	12 (1.04)	0.3441	12 (0.62)	11 (0.84)	0.4657
Lipid-lowing drugs	140 (5.41)	36 (5.54)	0.8986	134 (5.76)	57 (6.20)	0.6303	97 (4.67)	70 (6.05)	0.0880	101 (5.22)	77 (5.87)	0.4221
**Laboratory examination**												
TG (mg/dL)	66.38 (55.76–76.00)	69.03 (57.52–79.65)	0.0031	99.12 (87.62–111.51)	100.01 (89.39–113.28)	0.0246	136.29 (119.48–155.76)	138.06 (120.36–157.53)	0.1134	212.40 (176.12–271.70)	221.25 (179.66–285.86)	0.0027
TC(mg/dl)	158.12 (135.70–182.86)	158.51 (136.08–181.70)	0.9872	171.26 (148.84–196.00)	174.36 (149.23–196.39)	0.3158	180.54 (157.35–208.76)	183.25 (169.67–209.15)	0.2714	194.46 (168.94–225.77)	195.23 (170.10–223.45)	0.9685
HDL(mg/dl)	48.71 (40.98–58.38)	46.39 (39.82–54.51)	<0.0001	46.01 (38.27–54.90)	44.07 (38.27–52.58)	0.0024	44.07 (36.73–51.80)	42.53 (36.34–51.03)	0.0382	41.37 (35.18–49.48)	40.98 (35.18–49.10)	0.8380
LDL(mg/dl)	91.62 (73.07–11.34)	95.49 (76.93–115.21)	0.0074	104.00 (85.44–124.49)	107.86 (88.53–127.58)	0.0006	110.57 (88.92–133.76)	113.66 (92.40–134.54)	0.0210	114.43 (91.62–139.56)	114.43 (93.17–139.18)	0.4526
FBG (mg/dL)	86.58 (79.20–95.40)	88.20 (80.10–95.40)	0.0912	92.88 (84.60–102.60)	91.98 (84.42–102.60)	0.1875	102.6 (91.3–121.9)	103.1 (90.9–121.1)	0.5167	108.18 (95.04–136.80)	108.00 (95.22–132.48)	0.4949

*TYG index, triglyceride glucose index; NIHSS, National Institutes of Health Stroke Scale; BMI, body mass index; TG, Triglyceride; FBG, Fasting blood glucose; TC, total cholesterol; HDL, high density lipoprotein; LDL, low density lipoprotein; Q1–Q4, TYG index quartiles; SD, standard deviation; IQR, interquartile range*.

### Association of BMI With Outcome

One thousand seventy-nine patients died in the 12 months following their initial stroke occurrence, including 832 (9.32%) overweight/obese and 247 (6.12%) low/normal weight patients. The prognosis of patients with ischemic stroke at 12 months in each of the BMI categories was shown in Table 2. Compared to patients classified as low/normal weight, overweight/obese patients had a lower risk of death at 12 months after adjusting for potential covariates (9.32 vs. 6.12%; adj. HR 0.847, CI 95% 0.732–0.981). No significant association was found between being overweight/obese and poor functional outcome or stroke recurrence (*p* = 0.2738 and 0.3078). There was an interaction effect of age group by BMI for the risk of stroke or death (*p* = 0.0431 and 0.0173, respectively; Supplementary Table 1). An inverse relationship between BMI and stroke mortality (obesity paradox) was observed in patients older than 65 years, whereas no association between them was observed in young patients.

**Table 2:** Outcomes at 12 months follow up of ischemic stroke, stratified by BMI.

**Outcome**	**BMI**	**n (%) of Events**	**Crude HR/OR (95% CI)**	Adjusted HR/OR (95% CI)^*^	***P-*Value**
Stroke recurrence	BMI <25 kg/m^2^	609 (6.82)	1	1	0.3078
	BMI ≥ 25 kg/m^2^	286 (7.08)	0.6219 (0.900–1.912)	1.079 (0.932–1.248)	
^a^Poor outcome	BMI <25 kg/m^2^	2,064 (23.12)	1	1	0.2738
	BMI ≥ 25 kg/m^2^	749 (18.55)	0.757 (0.690–0.832)	0.941 (0.843–1.049)	
Death	BMI <25 kg/m^2^	832 (9.32)	1	1	0.0269
	BMI ≥ 25 kg/m^2^	247 (6.12)	0.647 (0.562–0.746)	0.847 (0.732–0.981)	

*TYG index ln[fasting triglycerides (mg/dl) × fasting glucose(mg/ dl)/2]; BMI [weight in kilograms divided by height in meters squared]*.

* *adjusted for sex, age, NIHSS score at admission, IV thrombolysis, smoking, medical history (Diabetes, Myocardial infarction, Atrial fibrillation, Hypertension, Hyperlipidemia), medication (Antihypertensive drugs, Antiplatelet drugs, Anticoagulation drugs, Lipid-lowing drugs, Hypoglycemic drugs), Laboratory examination (TG, TC, HDL, LDL, FBG)*.

a *Poor outcome, modified Rankin Scale score 3–6*.

### Association Between BMI and Stroke Outcome in Patients Stratified by TYG Index

**Figure 2:**
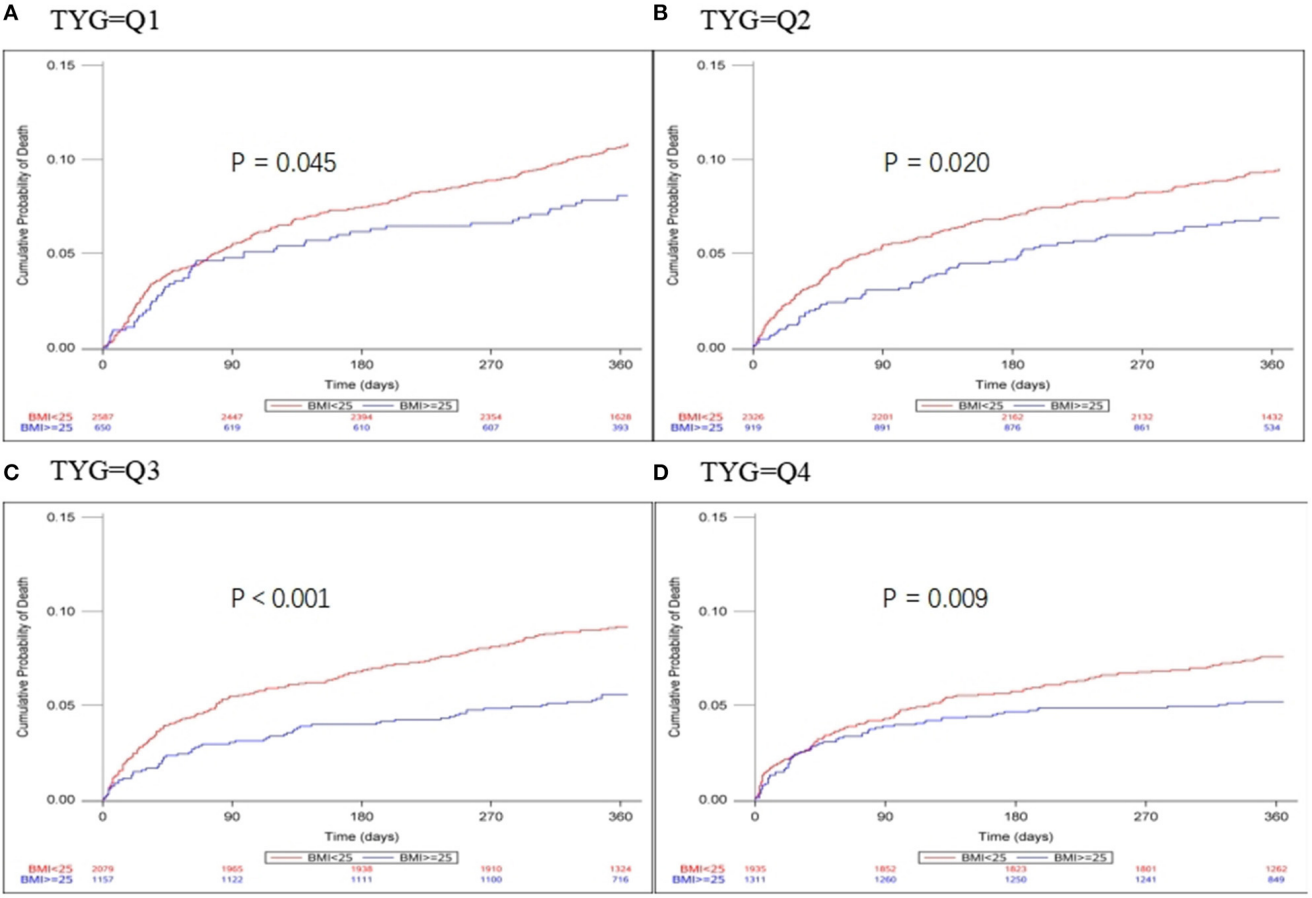
Kaplan-Meier time-to-event curves and log-rank tests for 1-year mortality in each BMI group, stratified by TYG index. **(A–D)** represent TYG = Q1, TYG = Q2, and TYG = Q3, respectively.

Patients were divided into quartiles according to TYG index: Q1 (TYG <8.33), Q2 (TYG 8.34–8.73), Q3 (TYG8.74–9.20), Q4 (TYG>9.21). Overweight and obese patients had a lower risk of death in all groups (Q1, crude HR 0.740, CI 95% 0.550–0.995; Q2, crude HR 0.717, CI 95% 0.542–0.948; Q3, crude HR 0.594 CI 95% 0.448–0.789; Q4, crude HR 0.683 CI 95% 0.512–0.910; Table 3, Figure 2), with no significant correlation after adjusting for all potential covariates (Q1, adj HR 0.892, CI 95% 0.658–1.208; Q2, adj HR 0.859, CI 95% 0.643–1.147; Q3, adj HR 0.827 CI 95% 0.617–1.107; Q4, adj HR 0.853 CI 95% 0.613–1.154). Similar results were found for the endpoints indicating a poor outcome. No significant association was found between BMI and stroke recurrence in the four groups. There was also no interaction between BMI and TYG index on the risk of stroke recurrence (*p* = 0.2449), mortality (*p* = 0.7887) and poor functional outcome (*p* = 0.7452; Table 3). In the analyses of continuous TyG index, BMI was associated with stroke mortality (adj HR 0.841, CI 95% 0.725–0.974, *p* = 0.0210), and there was no interaction between TYG index and BMI on stroke outcome (Table 4). Multivariate analysis of stroke outcomes by BMI and TYG index showed that overweight and obese patients had a lower risk of death at 12 months, and TYG index was associated with poor stroke outcomes (*p* = 0.0237, *p* = 0.0260; Table 5).

**Table 3:** Association between BMI and stroke outcomes stratified by TYG index.

**Outcome**	**TYG index**	**n (%) of Events**		**Crude HR/OR (95% CI)**	Adjusted HR/OR (95% CI)^*^	***P-*Value**	***P-*Value for interaction**
		**BMI <25 kg/m^**2**^**	**BMI ≥ 25 kgm^**2**^**				
Stroke recurrence	Q1	173 (6.69)	55 (8.46)	1.280 (0.945–1.733)	1.333 (0.974–1.824)	0.0726	0.2449
	Q2	153 (6.58)	60 (6.53)	0.987 (0.732–1.330)	1.049 (0.772–1.425)	0.7593	
	Q3	135 (6.49)	75 (6.48)	0.992 (0.748–1.315)	1.083 (0.810–1.450)	0.5895	
	Q4	148 (7.65)	96 (7.32)	0.952 (0.737–1.231)	0.962 (0.735–1.258)	0.7758	
^a^Poor outcome	Q1	675 (26.09)	140 (21.54)	0.778 (0.632–0.956)	0.997 (0.785–1.267)	0.9819	0.7887
	Q2	546 (23.47)	194 (21.11)	0.872 (0.725–1.050)	0.981 (0.790–1.219)	0.8651	
	Q3	472 (22.70)	211 (18.24)	0.759 (0.634–0.910)	0.905 (0.733–1.118)	0.3546	
	Q4	371 (19.17)	204 (15.56)	0.777 (0.644–0.937)	0.958 (0.768–1.196)	0.7042	
Death	Q1	277 (10.71)	52 (8.00)	0.740 (0.550–0.995)	0.892 (0.658–1.208)	0.4590	0.7542
	Q2	219 (9.42)	63 (6.86)	0.717 (0.542–0.948)	0.859 (0.643–1.147)	0.3021	
	Q3	190 (9.14)	64 (5.53)	0.594 (0.448–0.789)	0.827 (0.617–1.107)	0.2015	
	Q4	146 (7.55)	68 (5.19)	0.683 (0.512–0.910)	0.853 (0.631–1.154)	0.3039	

*TYG index ln[fasting triglycerides (mg/dl) × fasting glucose(mg/ dl)/2]; BMI [weight in kilograms divided by height in meters squared]*.

* *adjusted for sex, age, NIHSS score at admission, IV thrombolysis, smoking, medical history (Diabetes, Myocardial infarction, Atrial fibrillation, Hypertension, Hyperlipidemia), medication (Antihypertensive drugs, Antiplatelet drugs, Anticoagulation drugs, Lipid-lowing drugs, Hypoglycemic drugs), Laboratory examination (TG, TC, HDL, LDL, FBG)*.

a *Poor outcome, modified Rankin Scale score 3–6*.

**Table 4:** Influence of TYG index as a continuous variable on the relationship between BMI and stroke.

**Outcome**	**BMI**	**Crude HR/OR (95% CI)**	Adjusted HR/OR (95% CI)^*^	***P-*Value**	***P-*Value for interaction**
Stroke recurrence	BMI <25 kg/m^2^	1	1	0.4270	0.2529
	BMI ≥ 25 kg/m^2^	1.026 (0.890–1.184)	1.061 (0.916–1.229)		
^a^Poor outcome	BMI <25 kg/m^2^	1	1	0.3935	0.9176
	BMI ≥ 25 kg/m^2^	0.798 (0.726–0.877)	0.953 (0.854–1.064)		
Death	BMI <25 kg/m^2^	1	1	0.0210	0.9359
	BMI ≥ 25 kg/m^2^	0.680 (0.589–0.785)	0.841 (0.725–0.974)		

*TYG index ln[fasting triglycerides (mg/dl) × fasting glucose(mg/ dl)/2]; BMI [weight in kilograms divided by height in meters squared]*.

* adjusted for sex, age, NIHSS score at admission, IV thrombolysis, smoking, medical history (Diabetes, Myocardial infarction, Atrial fibrillation, Hypertension, Hyperlipidemia), medication (Antihypertensive drugs, Antiplatelet drugs, Anticoagulation drugs, Lipid-lowing drugs, Hypoglycemic drugs), Laboratory examination (TG, TC, HDL, LDL, FBG).

a *Poor outcome, modif*.

**Table 5:** Multivariate analysis of BMI and TYG index on stroke outcome.

**Variable**	**Stroke recurrence**		^****a****^**Poor outcome**		**Death**	
	**HR (95% CI)**	***P-*Value**	**OR (95% CI)**	***P-*Value**	**HR (95% CI)**	***P-*Value**
BMI≥25 kg/m^2^	1.065 (0.920–1.233)	0.4001	0.954 (0.854–1.064)	0.3972	0.844 (0.728–0.978)	0.0237
TYG index Q2	0.974 (0.803–1.180)	0.7844	0.971 (0.849–1.111)	0.6682	1.059 (0.897–1.249)	0.4982
TYG index Q3	0.970 (0.794–1.185)	0.7648	0.930 (0.806–1.072)	0.3158	1.038 (0.870–1.239)	0.6770
TYG index Q4	1.176 (0.957–1.446)	0.1228	0.838 (0.717–0979)	0.0262	1.071 (0.879–1.304)	0.4954
Age	1.022 (1.015–1.028)	<0.0001	1.070 (1.065–1.076)	<0.0001	1.072 (1.065–1.079)	<0.0001
Male,	1.009 (0.853–1.193)	0.9167	1.117 (0.990–1.260)	0.0715	0.957 (0.826–1.109)	0.5578
NHISS score median (IQR)	1.043 (1.034–1.053)	<0.0001	1.172 (1.160–1.830)	<0.0001	1.091 (1.085–1.098)	<0.0001
thrombolysis	0.867 (0.509–1.475)	0.5980	1.066 (0.742–1.531)	0.7303	0.697 (0.423–1.149)	0.1571
Prior/current smoker (%)	1.067 (0.907–1.256)	0.4330	0.953 (0.847–1.071)	0.4180	1.014 (0.876–1.174)	0.8519
Myocardial infarction	1.559 (1.088–2.233)	0.0156	1.038 (0.755–1.427)	0.8171	1.413 (0.996–2.004)	0.0526
Atrial fibrillation	1.804 (1.475–2.206)	<0.0001	1.211 (1.025–1.454)	0.0250	1.804 (1.475–2.206)	<0.0001
Hypertension	1.365 (1.138–1.637)	0.0008	1.292 (1.132–−1.474)	0.0001	1.365 (1.138–1.637)	0.0008
Hyperlipidemia	1.047 (0.762–1.438)	0.7767	0.685 (0.526–0.892)	0.0050	1.047 (0.762–1.438)	0.7767
Antihypertensive drugs	0.897 (0.755–1.065)	0.2141	0.827 (0.727–0.941)	0.0039	0.897 (0.755–1.065)	0.2141
Antiplatelet drugs	1.147 (0.958–1.347)	0.1347	1.303 (1.140–1.490)	0.0001	1.147 (0.958–1.374)	0.1347
Anticoagulation drugs	0.928 (0.506–1.704)	0.8096	1.214 (0.753–1.957)	0.4258	0.928 (0.506–1.704)	0.8096
Lipid-lowing drugs	0.977 (0.647–1.475)	0.9115	1.348 (0.965–1.881)	0.0796	0.977 (0.647–1.475)	0.9115
TC(mg/dl)	1.001 (0.999–1.003)	0.4254	1.001 (0.999–1.002)	0.5651	1.001 (0.999–1.003)	0.4254
HDL(mg/dl)	1.003 (0.998–1.007)	0.2232	0.999 (0.996–1.003)	0.6850	1.003 (0.998–1.007)	0.2232
LDL(mg/dl)	1.000 (0.998–1.002)	0.9268	1.001 (1.000–1.003)	0.0891	1.000 (0.998–−1.002)	0.9268

*TYG index ln[fasting triglycerides (mg/dl) × fasting glucose(mg/ dl)/2]; BMI [weight in kilograms divided by height in meters squared]*.

a *Poor outcome, modified Rankin Scale score 3–6*.

## Discussion

We found that overweight/obese stroke patients had a remarkably lower mortality compared to patients with normal/low BMI. There is an interaction between age and BMI. We found that association between BMI and stroke outcome was modified by age. However, we found no difference in outcomes in overweight/obese and normal/low-weight stroke patients that were stratified by TYG index and we did not observe an association between BMI and TYG index in ischemic stroke patients. A previous study examined IR using HOMA-IR suggested that the obesity paradox for mortality exists in insulin-resistant patients but not insulin-sensitive patients (6), suggesting IR may be part of the underlying mechanism of the obesity-stroke paradox. In this study, however, we did not observe a relationship between IR and the obesity-stroke paradox. Possible explanations for this result include that obesity is a fundamental risk factor for the onset and development of insulin resistance, and the majority of the population with IR is obese or overweight. IR results in excessive release of free fatty acids (FFAs) into circulation by unrestrained lipolysis (23). The increased flux of FFAs increases triglyceride synthesis in the liver and the synthesized triglyceride is then released into plasma, which raises the risk of hypertriglyceridemia and subsequent metabolic syndrome (23). When the storage capacity of adipose tissue reaches full capacity, the energy surplus presenting in obese patients leads to an increased efflux of FFAs and an ectopic accumulation of fat in the liver (24). As such, numerous studies have demonstrated that the TYG index is highly associated with the incidence of non-alcoholic fatty liver disease (NAFLD) (25–27). An epidemiological survey of a Chinese population also found that an elevation of the TYG index might predict an increased incidence of NAFLD (28). A high TYG index is more likely to reflect metabolic abnormalities after the occurrence of the IR. Previous studies have also shown that visceral fat (VAT) rather than subcutaneous fat (SAT) is correlated with the occurrence of cardiovascular diseases (29). Kim et al. found that a low VAT proportion is associated with favorable and excellent outcomes in acute ischemic stroke patients treated with intravenous thrombolysis (30). Another study found that better clinical outcomes in obese patients are associated with a lower proportion of VAT (30). Conversely, higher visceral and liver fat amounts have been found in IR obesity (31), suggesting that IR may counteract the paradoxically protective effects of obesity in stroke patients. Second, the presence of metabolically healthy obese (MHO) individuals, a previously identified subset of obese subjects without metabolic abnormalities (including IR and a proatherogenic lipoprotein profile) may also be responsible for the controversial results. Although MHO criteria have not been clearly defined, normal glucose and lipid metabolism parameters, in addition to the absence of hypertension, is considered typical MHO diagnostic criteria (32). For example, Sánchez-Iñigo et al. found that metabolically unhealthy obese/non-obese (MUNO/MUO) individuals exhibit a greater risk of ischemic stroke than metabolically healthy non-obese, in which the TYG index is used to define a metabolically healthy state (MUNO, HR 1.55, CI 95% 1.36–1.77; MUO, HR 1.86, CI 95% 1.57–2.21) (33). Last, TYG index does not fully represent IR. Many studies have suggested that TYG index can be used as a surrogate of insulin resistance in otherwise healthy-appearing patients (15, 21, 34, 35). However, these findings lack consistency and a systematic review deemed the evidence for the usefulness of the TYG index as a surrogate biochemical marker of IR as “moderate-to-low quality.”

We identified the obesity paradox in all four groups before the covariates were adjusted, but the results were no longer significant after adjustment. Indeed, whether or not the obesity paradox is a true phenomenon is still the subject of much debate. Some have proposed that the paradox is caused by physiological factors that reduce risk in obese participants (36), whereas others suggested that it is an artifact resulting from selection bias in observational studies (37). From clinical and epidemiological observations, many contributing factors have been identified that lead to lower mortality, such as demographic characteristics (e.g., age, sex, educational status) and physical conditions (e.g., severity and duration of heart disease, initial neurological severity, and cachexia). In the present study, we found that overweight/obese patients are more likely to use antihypertensive, lipid-lowering, and antiplatelet drugs at baseline. We adjusted for patient characteristics that include sex, age, NIHSS score at admission, IV thrombolytic therapy, smoking, medical history (i.e., myocardial infarction, atrial fibrillation, hypertension, hyperlipidemia), and medication (i.e., antihypertensive, antiplatelet, anticoagulation, lipid-lowing, and hypoglycemic drugs). Despite the adjustments for these potentially confounding factors, the obesity paradox remained, with an adjusted HR for overall mortality in the overweight/obese group being 0.847 (0.732–0.981). A previous study including a cohort of 21,884 patients from the Danish National Indicator Project registry, found that post-stroke mortality in the 5-year follow-up period is lower in overweight (BMI 25.0–29.9 kg/m^2^) and obese (BMI 30.0–34.9 kg/m^2^) patients compared to those with normal weight (BMI 18.5–24.9 kg/m^2^) and underweight (BMI <18.5 kg/m^2^) (5). We also identified a BMI-age interaction since obese or overweight stroke patients older than 65 have a lower mortality rate, while the interaction is not presented in patients younger than 65. In contrast to our results, Vemmos et al. found that the protective effect of BMI on mortality decreases with increasing age (3). However, a *post-hoc* analysis of a randomized controlled trial in China (Clopidogrel in High-risk patients with Acute Non-disabling Cerebrovascular Events) did not identify the obesity paradox or observe an influence of age on the association of weight status with the prognosis of stroke (38). In a national wide representative survey of U.S. adults, an effect of age among stroke survivors was discovered, whereby a higher BMI was associated with a marginally increased mortality in younger individuals that reduced linearly with increasing age. This is similar to our findings (39).

We acknowledge several limitations with our findings. In particular, as a *post-hoc* analysis, several variables of interest were not accessible for analysis because they were not recorded. Additionally, a comparison of BMI with the waist-to-hip ratio cannot be evaluated. Our findings also use baseline BMI, fasting triglyceride and glucose, which may have confounding effects on the results as the collection of consecutive data about body weight, fasting glucose, and triglyceride at the 12 months follow-up is unavailable. Measurement errors across multiple centers may also slightly impact patients' fasting triglyceride and glucose levels, yet the laboratory values of these studies should still be comparable as the measurement techniques in all centers are based on the recommendation of the International Federation of Clinical Chemistry and Laboratory Medicine. A final limitation of our study is the relatively short follow-up period of 12 months. Several other studies have tracked patients much longer. For example, Korean Stroke Registry included 2,317 patients with ischemic stroke with 7.5-year follow-up (40) and the Prospective investigation of Greece included 2,785 first-ever acute stroke patients with a 10-year follow up (3).

In conclusion, we found that overweight and obese stroke patients have significantly lower mortality compared to patients with normal BMI, in line with previous reports of the stroke obesity paradox. Additionally, our findings suggest that age may influence the relationship between BMI and stroke prognosis. Stratification by TYG index eliminated the presence of the obesity paradox in this patient cohort, and thus TYG index, or its correlate IR, may not be beneficial in understating the metabolic mechanisms responsible for the stroke obesity paradox.

## Data Availability Statement

The data analyzed in this study is subject to the following licenses/restrictions: The datasets generated for this study are available on request to the corresponding author. Requests to access these datasets should be directed to YW, yongjunwang@ncrcnd.org.cn.

## Ethics Statement

The studies involving human participants were reviewed and approved by The Central Institutional Review Board at Beijing Tiantan Hospital. The patients/participants provided their written informed consent to participate in this study.

## Author Contributions

ZH, YP, YY, XY, and YoW contributed to the conception and design of the study. YiW and XZ assisted with data acquisition and interpretation. ZL and XM coordinated the study. YP and XX conducted the statistical analysis. ZH contributed to drafting. YoW is the guarantor for this paper. All authors read and approved the final manuscript.

## Conflict of Interest

The authors declare that the research was conducted in the absence of any commercial or financial relationships that could be construed as a potential conflict of interest.

## References

[1] Feigin VL Forouzanfar MH Krishnamurthi R Mensah GA Connor M Bennett DA. Global and regional burden of stroke during 1990-2010: findings from the global burden of disease study 2010. Lancet. (2014) 383:245–54. 10.1016/S0140-6736(13)61953-4 24449944 PMC4181600

[2] Bazzano LA Gu D Whelton MR Wu X Chen CS Duan X. Body mass index and risk of stroke among Chinese men and women. Ann Neurol. (2010) 67:11–20. 10.1002/ana.21950 20186847 PMC4371851

[3] Vemmos K Ntaios G Spengos K Savvari P Vemmou A Pappa T. Association between obesity and mortality after acute first-ever stroke: the obesity-stroke paradox. Stroke. (2011) 42:30–6. 10.1161/STROKEAHA.110.593434 21127299

[4] Oesch L Tatlisumak T Arnold M Sarikaya H. Obesity paradox in stroke - Myth or reality? A systematic review. PLoS ONE. (2017) 12: e0171334. 10.1371/journal.pone.0171334 28291782 PMC5349441

[5] Olsen TS Dehlendorff C Petersen HG Andersen KK. Body mass index and poststroke mortality. Neuroepidemiology. (2008) 30: 93–100. 10.1159/000118945 18309248

[6] Xu J Wang A Meng X Jing J Wang Y Wang Y. Obesity-stroke paradox exists in insulin-resistant patients but not insulin sensitive patients. Stroke. (2019) Strokeaha118023817. 10.1161/strokeaha.118.023817. [Epub ahead of print]. 31043152

[7] Kernan WN Inzucchi SE Viscoli CM Brass LM Bravata DM Horwitz RI. Insulin resistance and risk for stroke. Neurology. (2002) 59:809–15. 10.1212/WNL.59.6.809 12349850

[8] Martinez KE Tucker LA Bailey BW LeCheminant JD. Expanded normal weight obesity and insulin resistance in US adults of the national health and nutrition examination survey. J Diabetes Res. (2017) 2017:9502643. 10.1155/2017/9502643 28812029 PMC5547730

[9] Ago T Matsuo R Hata J Wakisaka Y Kuroda J Kitazono T. Insulin resistance and clinical outcomes after acute ischemic stroke. Neurology. (2018) 90:e1470–7. 10.1212/WNL.0000000000005358 29602916

[10] Jing J Pan Y Zhao X Zheng H Jia Q Mi D. Insulin resistance and prognosis of nondiabetic patients with ischemic stroke: the ACROSS-China study (Abnormal glucose regulation in patients with acute stroke across China). Stroke. (2017) 48:887–93. 10.1161/STROKEAHA.116.015613 28235959

[11] Deng XL Liu Z Wang C Li Y Cai Z. Insulin resistance in ischemic stroke. Metab Brain Dis. (2017) 32:23–34. 10.1007/s11011-017-0050-0 28634787

[12] Fitzgibbons TP Czech MP. Emerging evidence for beneficial macrophage functions in atherosclerosis and obesity-induced insulin resistance. J Mol Med. (2016) 94:267–75. 10.1007/s00109-016-1385-4 26847458 PMC4803808

[13] DeFronzo RA Tobin JD Andres R. Glucose clamp technique: a method for quantifying insulin secretion and resistance. Am J Physiol. (1979) 237:E214–23. 10.1152/ajpendo.1979.237.3.E214 382871

[14] Li S Yin C Zhao W Zhu H Xu D Xu Q. Homeostasis model assessment of insulin resistance in relation to the poor functional outcomes in nondiabetic patients with ischemic stroke. Biosci Rep. (2018) 38:3. 10.1042/BSR20180330 29588341 PMC5938425

[15] Simental-Mendía LE Rodríguez-Morán M Guerrero-Romero F. The product of fasting glucose and triglycerides as surrogate for identifying insulin resistance in apparently healthy subjects. Metab Synd Relat Disord. (2008) 6:299–304. 10.1089/met.2008.0034 19067533

[16] Mohd Nor NS Lee S Bacha F Tfayli H Arslanian S. Triglyceride glucose index as a surrogate measure of insulin sensitivity in obese adolescents with normoglycemia, prediabetes, and type 2 diabetes mellitus: comparison with the hyperinsulinemic-euglycemic clamp. Pediatr Diabetes. (2016) 17:458–65. 10.1111/pedi.12303 26251318

[17] Zhou Y Pan Y Yan H Wang Y Li Z Zhao X. Triglyceride glucose index and prognosis of patients with ischemic stroke. Front Neurol. (2020) 11:456. 10.3389/fneur.2020.00456 32587566 PMC7297915

[18] Li Z Wang C Zhao X Liu L Wang C Li H. Substantial progress yet significant opportunity for improvement in stroke care in China. Stroke. (2016) 47:2843–9. 10.1161/STROKEAHA.116.014143 27758941

[19] Wang Y Cui L Ji X Dong Q Zeng J Wang Y. The China National Stroke Registry for patients with acute cerebrovascular events: design, rationale, and baseline patient characteristics. Int J Stroke. (2011) 6:355–61. 10.1111/j.1747-4949.2011.00584.x 21609414

[20] Obesity: Preventing and Managing the Global Epidemic. Report of a WHO Consultation. Geneva: World Health Organization Technical Report Series (2000). 11234459

[21] Guerrero-Romero F Simental-Mendía LE González-Ortiz M Martínez-Abundis E Ramos-Zavala MG Hernández-González SO. The product of triglycerides and glucose, a simple measure of insulin sensitivity. Comparison with the euglycemic-hyperinsulinemic clamp. J Clin Endocrinol Metab. (2010) 95:3347–51. 10.1210/jc.2010-0288 20484475

[22] Gu HQ Li ZX Zhao XQ Liu LP Li H Wang CJ. Insurance status and 1-year outcomes of stroke and transient ischaemic attack: a registry-based cohort study in China. BMJ Open. (2018) 8:e021334. 10.1136/bmjopen-2017-021334 30068612 PMC6074626

[23] Neuhauser HK. The metabolic syndrome. Lancet. (2005) 366:1922–3; author reply 1923–4. 10.1016/S0140-6736(05)67782-3 16325690

[24] Donnelly KL Smith CI Schwarzenberg SJ Jessurun J Boldt MD Parks EJ. Sources of fatty acids stored in liver and secreted via lipoproteins in patients with nonalcoholic fatty liver disease. J Clin Invest. (2005) 115:1343–51. 10.1172/JCI23621 15864352 PMC1087172

[25] Kitae A Hashimoto Y Hamaguchi M Obora A Kojima T Fukui M. The triglyceride and glucose index is a predictor of incident nonalcoholic fatty liver disease: a population-based cohort study. Can J Gastroenterol Hepatol. (2019) 2019:5121574. 10.1155/2019/5121574 31687367 PMC6800935

[26] Lee SB Kim MK Kang S Park K Kim JH Baik SJ. Triglyceride glucose index is superior to the homeostasis model assessment of insulin resistance for predicting nonalcoholic fatty liver disease in Korean adults. Endocrinol Metab. (2019) 34:179–86. 10.3803/EnM.2019.34.2.179 31257745 PMC6599902

[27] Zhang S Du T Zhang J Lu H Lin X Xie J. The triglyceride and glucose index (TyG) is an effective biomarker to identify nonalcoholic fatty liver disease. Lipids Health Dis. (2017) 16:115. 10.1186/s12944-017-0409-6 28103934 PMC5248473

[28] Zheng R Du Z Wang M Mao Y Mao W. A longitudinal epidemiological study on the triglyceride and glucose index and the incident nonalcoholic fatty liver disease. Lipids Health Dis. (2018) 17:1262. 10.1186/s12944-018-0913-3 30458848 PMC6247753

[29] Neeland IJ Poirier P Després JP. Cardiovascular and metabolic heterogeneity of obesity: clinical challenges and implications for management. Circulation. (2018) 137:1391–406. 10.1161/CIRCULATIONAHA.117.029617 29581366 PMC5875734

[30] Kim JH Choi KH Kang KW Kim JT Choi SM Lee SH. Impact of visceral adipose tissue on clinical outcomes after acute ischemic stroke. Stroke. (2019) 50:448–54. 10.1161/STROKEAHA.118.023421 30612535

[31] Kahn SE Hull RL Utzschneider KM. Mechanisms linking obesity to insulin resistance and type 2 diabetes. Nature. (2006) 444:840–6. 10.1038/nature05482 17167471

[32] Blüher M. Metabolically healthy obesity. Endocr Rev. (2020) 41:405–20. 10.1210/endrev/bnaa004 PMC7098708 32128581

[33] Sánchez-Iñigo L Navarro-González D Fernández-Montero A Pastrana-Delgado J Martínez JA. Risk of incident ischemic stroke according to the metabolic health and obesity states in the Vascular-Metabolic CUN cohort. Int J Stroke. (2017) 12:187–91. 10.1177/1747493016672083 28134052

[34] Almeda-Valdés P Bello-Chavolla OY Caballeros-Barragán CR Gómez-Velasco DV Viveros-Ruiz T Vargas-Vázquez A. [Índices para la evaluación de la resistencia a la insulina en individuos mexicanos sin diabetes]. Gac Med Mex. (2018) 154:(Suppl. 2):S50–5. 10.24875/GMM.18004578 30532124

[35] Qu C Zhou X Yang G Li L Liu H Liang Z. The natural logarithm of zinc-α2-glycoprotein/HOMA-IR is a better predictor of insulin sensitivity than the product of triglycerides and glucose and the other lipid ratios. Cytokine. (2016) 79:96–102. 10.1016/j.cyto.2015.12.024 26797477

[36] Kastorini CM Panagiotakos DB. The obesity paradox: methodological considerations based on epidemiological and clinical evidence–new insights. Maturitas. (2012) 72:220–4. 10.1016/j.maturitas.2012.04.012 22609156

[37] Banack HR Kaufman JS. The “obesity paradox” explained. Epidemiology. (2013) 24:461–2. 10.1097/EDE.0b013e31828c776c 23549182

[38] Chen W Pan Y Jing J Zhao X Liu L Meng X. Association of body mass index and risk of stroke after acute minor stroke or tia: a *post hoc* analysis of a randomized controlled trial. Neurotox Res. (2019) 36:836–43. 10.1007/s12640-019-00056-4 31127478

[39] Towfighi A Ovbiagele B. The impact of body mass index on mortality after stroke. Stroke. (2009) 40:2704–8. 10.1161/STROKEAHA.109.550228 19542056

[40] Choi KM Cho HJ Choi HY Yang SJ Yoo HJ Seo JA. Higher mortality in metabolically obese normal-weight people than in metabolically healthy obese subjects in elderly Koreans. Clin Endocrinol. (2013) 79:364–70. 10.1111/cen.12154 23330616

