# Supplementary material for: An Analysis of the Potential Relationship of Triglyceride Glucose and Body Mass Index With Stroke Prognosis
Source: Front Neurol. 2021 Apr 22;12:630140. doi: 10.3389/fneur.2021.630140 (PMC8101495; doi:10.3389/fneur.2021.630140)
Supplement: Supplementary file 1 [file Table_1.DOCX]

**Supplementary Table 1** **Outcomes at 12 months after ischemic stroke for each BMI category, classified by age.**

| Outcome | BMI | Age < 65 years | | | Age ≥ 65 years | | | P Value |
| --- | --- | --- | --- | --- | --- | --- | --- | --- |
|  |  | n (%) of Events | Adjusted HR / OR  (95% CI)* | P value | n (%) of Events | Adjusted HR / OR  (95% CI)* | P value |  |
| Stroke recurrence | BMI < 25 kg/㎡ | 189 (4.73) | 1 | 0.0630 | 420 (8.52) | 1 | 0.5011 | 0.0431 |
|  | BMI ≥ 25 kg/㎡ | 141 (6.11) | 1.237  (0.989-1.549) |  | 145 (8.38) | 0.936  (0.770-1.136) |  |  |
| ^a^Poor outcome | BMI < 25 kg/㎡ | 460 (11.50) | 1 | 0.1820 | 1604 (32.56) | 1 | 0.0628 | 0.8444 |
|  | BMI ≥ 25 kg/㎡ | 234 (10.15) | 0.883  (0.736-1.060) |  | 515 (29.75) | 0.881  (0.772-1.007) |  |  |
| Death | BMI < 25 kg/㎡ | 126 (3.15) | 1 | 0.6932 | 706 (14.33) | 1 | 0.0001 | 0.0173 |
|  | BMI ≥ 25 kg/㎡ | 73 (3.17) | 1.062  (0.789-1.429) |  | 174 (10.05) | 0.717  (0.605-0.852) |  |  |

*Adjusted for sex, age, NIHSS score at admission, IV thrombolysis, smoking, medical history (Diabetes, Myocardial infarction, Atrial fibrillation, Hypertension, Hyperlipidemia), medication (Antihypertensive drugs, Antiplatelet drugs, Anticoagulation drugs, Lipid-lowing drugs, Hypoglycemic drugs), Laboratory examination (TG, TC, HDL, LDL, FBG)

a: Poor outcome, modified Rankin Scale score 3–6.
